# Supplementary material for: Conditional chemoconnectomics (cCCTomics) as a strategy for efficient and conditional targeting of chemical transmission
Source: eLife. 2024 Apr 30;12:RP91927. doi: 10.7554/eLife.91927 (PMC11060718; doi:10.7554/eLife.91927)
Supplement: Figure 3—source data 3. [file elife-91927-fig3-data3.zip › Figure 3 source data3 part 2.zip!/Figure 3-Source data 3-part2/1060f2-1-CRISPResso_Report_gl5umwyq.zip!/CRISPResso_on_1060f2_3/1c.Alignment_barplot.pdf]

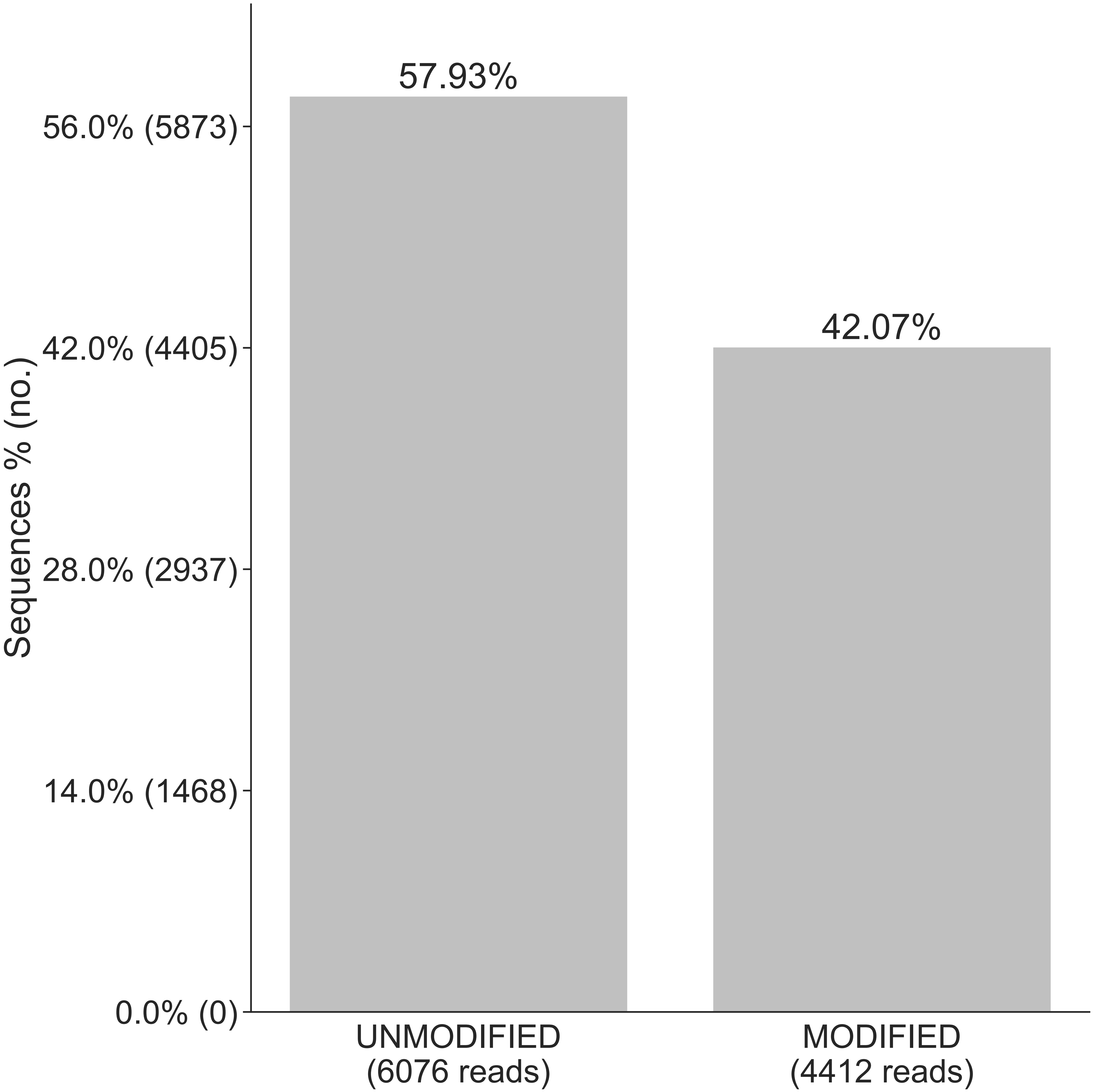
57.93%
42.07%
56.0% (5873)
42.0% (4405)
28.0% (2937)
14.0% (1468)
0.0% (0)
Sequences % (no.)
UNMODIFIED
(6076 reads)
MODIFIED
(4412 reads)
